# Evaluating the Role of Intermediate Screws in the Early Healing of Traumatic Thoracolumbar Fractures Managed by Short-Segment Fixation

**DOI:** 10.7759/cureus.82574

**Published:** 2025-04-19

**Authors:** Ritesh Runu, Santosh Kumar, Vaibhav Sanchay, Nishant Kashyap, Abhijeet Subhash

**Affiliations:** 1 Orthopedics, Indira Gandhi Institute of Medical Sciences, Patna, Patna, IND

**Keywords:** asia score, fracture healing duration, intermediate screws, kyphotic deformity, short-segment posterior fixation, spinal cord injury, thoracolumbar fracture, wedge compression fracture

## Abstract

Introduction

Thoracolumbar (TL) fracture is one of the leading problems in orthopedic practice, more so in the modern era, where individuals are more at risk due to high-energy trauma. By definition, the thoracolumbar region of the spine spans from T11 to L2. The TL region accounts for around 60%-70% of all traumatic spinal fractures. The instrumentation of this region is still highly debatable, but in the modern era, posterior short-segment transpedicular screw fixation, which offers shorter operating times, less intraoperative blood loss, and better motion preservation is preferred for fixing these fractures over long-segment fixation (LSF). Intermediate screw fixation at the fractured vertebrae provides stability without compromising the mobility of the spine.

Methods

A prospective observational study was undertaken in the Department of Orthopedics of Indira Gandhi Institute of Medical Sciences, Patna, from August 2022 to March 2024. Thirty thoracolumbar injury patients who gave consent for surgery, admitted during the study period, were included in the study. After surgical fitness, patients underwent the operation. Polyaxial pedicle screws were inserted using intersection technique; first, screws were inserted above and below the fractured vertebrae and then in the fractured pedicle after checking the pedicle morphology. Clinical examination and neurological charting per the American Spinal Injury Association (ASIA) Impairment Scale (AIS) were done during admission and follow-up. Radiological examinations were done for all patients. Patients were followed up at six weeks and three, six, and 12 months.

Results

A total of 30 patients (20 male and 10 female patients) were included in our study. The mean age of the patients was 32.9 (range: 18-60) years. The most common cause of thoracolumbar injuries was fall from height (23, 77%); other causes include road traffic accidents (RTAs) (7, 23%). The most common vertebra involved in this study was L1 (12, 40%); other involved the D12 and L2 vertebrae. The most common pattern was compression fracture (21, 70%), followed by burst fracture (9, 30%). The mean duration from injury to hospital admission was 8.33±10.73 days. The mean duration from injury to surgery was 17.2±11.02 days. The mean duration of hospital stay was 22.9±6.1 days. Six (20%) thoracolumbar fracture patients were operated on within 10 days of injury, and 24 (80%) were operated on after 10 days of injury. Preoperatively, there were four (13.33%) AIS grade A patients, 18 (60%) AIS B, four (13.33%) AIS C, four (13.33%) AIS D, and 0 (0%) AIS E. At the end of one year, there were four (13.33%) AIS A patients, 0 (0%) AIS B, 0 (0%) AIS C, 14 (46.67%) AIS D, and 12 (40%) AIS E. Preoperatively, the mean kyphotic angles were 22.6°±1.2°; at one year, the mean kyphotic angle was 8.1°±1.1°. The mean difference between the preoperative and one-year postoperative period was 14.5°. The p-value was less than 0.0005; there was a significant difference between preoperative and postoperative kyphotic angles. In this study, of 30 patients, 23 (77%) had no complications, three (10%) had pressure sores, two (3.33%) had discharge from the incision site, and two (3.33%) had urinary tract infection (UTI).

Conclusion

Introducing intermediate screw in short-segment fixation (SSF) provides significant improvement in mean kyphotic angle and early signs of fracture healing without implant failure. The mobility of the spine remains intact.

## Introduction

By definition, the thoracolumbar (TL) region of the spine spans from T11 to L2. The TL region accounts for around 60%-70% of all traumatic spinal fractures [1]. Traumatic spinal cord injury occurs in an estimated 29-50 cases per million population per year worldwide. The annual incidence of TL fractures is about 30 per 100,000 inhabitants if osteoporotic fractures are counted together [2].

These injuries can result in loss of neurological function, pain, disability, and deformity of the spine and represent a great economic burden to society. The management and care of spinal cord injury patients is labor-intensive, costly, and highly demanding, with prolonged bed occupancy. In resource-limited settings like ours, due to a lack of infrastructure and training, the majority of patients with spinal injury undergo conservative management, compromising their overall health. This underscores the role of surgical stabilization, early mobilization, and patient rehabilitation for better outcomes.

Thoracolumbar injuries are very common, and the risk of neurological injury is high due to the change from the rigid thoracic segment to the mobile lumbar segment and precarious blood supply in the TL region [3]. Therefore, these injuries require early decompression and rigid stabilization, which can be achieved by posterior long-segment fixation (LSF) [4]. The problem with LSF is prolonged surgery, increased blood loss, and spine rigidity [5]. Comparatively, short-segment fixation (SSF) requires shorter operative time, less blood loss, and maintained spinal mobility at the cost of instability [6,7]. Mahar et al. concluded that segmental fixation of burst fractures with screws at the level of the fracture offers improved biomechanical stability [8]. Theoretically, segmental fixation provides additional fixation points that may aid in fracture reduction and kyphosis correction. We hypothesized that intermediate screws help in the early healing of fracture vertebrae by transmission of load through screws and decreases chances of implant failure even after early mobilization and loss of correction of kyphosis. The aim of present study is to estimate the role of intermediate screws in traumatic thoracolumbar fractures managed by short-segment fixation.

## Materials and methods

This was a prospective study conducted in the Department of Orthopedics of a government medical college in eastern India from August 2022 to March 2024, after obtaining institutional ethics committee approval (605/IEC/IGIMS/2022) on July 1, 2022. The inclusion criteria were age 18-60 years and single-level thoracolumbar fracture of the spine with Thoracolumbar Injury Classification and Severity Score (TLICS) > 4 with or without bowel and bladder involvement. Patients who were operated previously, those operated at the same site who had local or generalized infection, those with chronic illness causing spinal instability, those not giving consent for the study, and those lost to follow-up were excluded. Those who met the inclusion criteria were followed up for one year. Clinical and neurological assessment was done using the American Spinal Injury Association (ASIA) Impairment Scale (AIS). Radiological evaluation with preoperative X-ray and magnetic resonance imaging (MRI) of the thoracolumbar spine was also conducted, as well as postoperative X-ray on postoperative day 3 (POD 3). Patients were followed up for one year with X-rays at six weeks, 12 weeks, six months, and one year.

Surgical technique

After preanesthetic checkup and obtaining informed consent, the patients were operated on under general anesthesia. On the operating table (OT), the patient was positioned prone over the bolster so that the spine is extended and the abdomen hangs freely. The injured vertebrae level was marked with an image intensifier, keeping it in the central area; painting and draping were done. The skin, subcutaneous tissue, and paraspinal muscles were infiltrated with 1:50,000 epinephrine solution to reduce the bleeding. A midline incision was taken up to two levels above and below the fractured vertebra. Paraspinal muscles were retracted, and facet joints were exposed. The pedicle entry point was marked using the intersection technique [9]. Then, a pedicle awl entry was made. A pedicle sound was inserted to ascertain the intact walls, and the length was measured. In male patients, 5.5 and 6.5 mm polyaxial screws were used, while in female patients, 4.5 and 5.5 mm were used, as per the built of the patient. Pedicle screws passed in both pedicles of upper and lower vertebrae, sparing the fractured vertebra. After short-segment fixation, an intermediate screw was passed in the fractured vertebra when the pedicle was found intact. The implant position was checked under an image intensifier television (IITV). Then, a rod was applied on one side. An image intensifier was used to verify the screw placements during insertion in both lateral and anteroposterior views. Unilateral laminectomy and cord decompression were performed, and the cord was inspected at the level of the injured vertebra. After adequate decompression, rods were tightened on both sides, hemostasis achieved. The spinous process was realigned and sutured. Drain was applied, sequential layer closure of the wound was done, and an occlusive dressing was applied.

Postoperatively, the patients were placed on IV fluids and administered IV analgesics and IV antibiotics for three days (second-generation cephalosporins (cefuroxime) and aminoglycoside). Supine position was maintained, and an input-output chart was maintained. On POD 1, the patients were turned to their sides; sitting was allowed with anterior hyperextension braces. Chest and limb physiotherapy was started. On POD 2, the dressing was changed, the drain was removed, and physiotherapy continued. Radiological and neurological parameters were carefully recorded as per protocol.

Statistical analysis

Data were entered into Microsoft Excel (Microsoft Corp., Redmond, WA) and analyzed using SPSS for Windows version 24.0 (SPSS Inc., Chicago, IL). Descriptive statistics were applied, with categorical variables expressed as frequencies and percentages, while continuous variables as mean and standard deviation (SD). A comparative analysis between groups was conducted using appropriate statistical tests.

## Results

A total of 30 patients (20 male and 10 female patients) were included in our study. The mean age of the patients was 32.9 (range: 18-60) years. The most common cause of injury in our study was fall from height (23, 77%) (fall from a tree, building, and stairs). Other causes include road traffic accidents (RTAs) (7, 23%). The commonest vertebral level injured was L1 (12, 40%); other levels were D12 (8, 27%), L2 (6, 20%), L3 (3, 10%), and L4 (1, 3%). The patients were assessed for neurological status, and AIS grading was used. Preoperative and postoperative AIS grading is mentioned in Table 1.

**Table 1:** AIS grading for neurological status (N=30) Data are represented as numbers and percentages. AIS grade was mentioned, and in further follow-up, patients' AIS grades improved as shown. This table shows that after adequate decompression, there was an improvement in the AIS grade. AIS A patients remained in AIS A after a one-year follow-up, showing that complete cord injuries do not heal completely, and residual paralysis is sustained. AIS: American Spinal Injury Association Impairment Scale, POD: postoperative day

AIS grade	A	B	C	D	E
Preoperative	4 (13.33%)	18 (60%)	4 (13.33%)	4 (13.33%)	0 (0%)
Postoperative (POD 3)	4 (13.33%)	18 (60%)	4 (13.33%)	4 (13.33%)	0 (0%)
6 weeks	4 (13.33%)	2 (6.67%)	12 (40%)	12 (40%)	0 (0%)
3 months	4 (13.33%)	0 (0%)	11 (36.67%)	9 (30%)	6 (20%)
6 months	4 (13.33%)	0 (0%)	3 (10%)	11 (36.67%)	12 (40%)
12 months	4 (13.33%)	0 (0%)	0 (0%)	14 (46.67%)	12 (40%)

The angular deformity of the spine was measured by Cobb's angle on X-ray. Preoperative and postoperative Cobb's angle is presented in Table 2.

**Table 2:** Loss of kyphotic correction KA: kyphotic angle, POD: postoperative day

Comparison	Mean difference	t-test	p-value
Preoperative KA versus postoperative (POD 3) KA	16.10	53.53	0.0003
Postoperative (POD 3) KA versus 6 week KA	-0.40	37.99	0.0003
Postoperative (POD 3) KA versus 3 month KA	-0.81	47.42	0.0003
Postoperative (POD 3) KA versus 6 month KA	-1.22	56.40	0.0003
Postoperative (POD 3) KA versus 12 month KA	-1.62	38.33	0.0003

As shown in Table 2, the mean correction of kyphosis during surgery was 16.10. Further loss of correction during follow-up is seen, but none was significant, and clinically, there was no deterioration of neurological recovery and implant failure.

**Figure 1:**
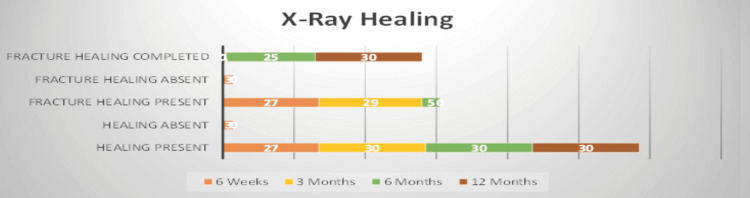
Graphical representation of fracture healing pattern The X-axis represents the duration of fracture healing. The Y-axis represents the stage of fracture healing.

Postoperative complication was seen in seven (23%) patients. Complications include pressure sores (3, 10%). All were in grade AIS A, which were managed by regular posture change every two hours and regular dressing. One case required secondary suturing. Discharge from the incision site occurred in two (7%) patients, which was managed by regular dressing, and they all healed within a week. Urinary tract infection (UTI) occurred in two (7%) patients due to long-standing indwelling urinary catheters, which was managed by appropriate antibiotics based on urine culture and sensitivity reports. None of the patients had implant failure. On X-ray, signs of healing include ossification of vertebral bodies, reduced lucent area in vertebral bodies, formation of trabecular patterns, and increased density of the anterior vertebral body cortex and interdiscal cortex. The duration of fracture healing is shown in Figure 1.

## Discussion

Short-segment fixation in TL fractures is increasingly used for single-level or double-level fractures. The introduction of intermediate screws is still controversial vis-a-vis stability and early mobilization. SSF has the limitation of rigidity of fixation and the risk of early failure on mobilization. We hypothesized that the introduction of intermediate screws helps in the transfer of body weight to the injured vertebra, which helps in the early consolidation of fractured vertebra and prevents loss of correction and implant failure. In our study of 30 patients with single-level TL fractures, L1 was the most common vertebra that was fractured. In the cadaveric study by Mahar et al., intermediate screws were used for biomechanical stability of construct [8]. Similar findings were noted by Guven et al. [10] and Bolesta et al. [11]. Patients were managed by posterior instrumentation and cord decompression with short-segment fixation along with intermediate screw fixation. The preoperative mean kyphotic angle was 22.6°±1.2°, which improved to 6.5°±1.1° postoperatively. This change was statistically significant. On further follow-up, the mean kyphotic angles at six weeks, 12 weeks, 24 weeks, and 12 months were 6.9°±1.1°, 7.3°±1.1°, 7.7°±1.1°, and 8.1°±1.1°, respectively.

There was progressive loss of correction of 0.39°, 0.42°, 0.39°, and 0.34° at six weeks, 12 weeks, 24 weeks, and 12 months, respectively. Effectively, there was minimal loss in kyphosis correction, which may be due to early consolidation of injured vertebrae along with good healing of soft tissue, effective bracing, and back extension exercises. This was also evident through radiological assessment, as shown in Figure 1. The early healing signs, i.e., ossification of vertebral bodies, reduced lucent area in vertebral bodies, and formation of trabecular patterns, were visible according to Wolff's law of bone healing. It dictates that the amount of bone healing is directly proportional to the amount of force across the fracture site. Early bone formation reduces the risk of implant loosening; hence, we did not face any cases of implant failures in our study. The AIS grading of spinal injury preoperatively was as follows: A, 4 (13.33%); B, 18 (60%); C, 4 (13.33 %); D, 4 (13.33%), and E, 0 (0%). Postoperatively and at one-year follow-up, there were four (13.33%) grade A patients, 0 (0%) grade B and C, 14 (46.67%) grade D, and 12 (40%) grade E. This shows that patients with grade A severe spinal cord injury had no recovery, and those with partial cord injury had good recovery collectively. This may be due to early fixation and adequate spine decompression.

Study limitations

Our study had a small number of patients, which was only 30, due to limited time and patient inclusion. Further studies with a greater number of patients should be conducted. Also, our study was non-randomized and included single-level TL injuries. Lastly, the use of normal X-rays to define the angle of kyphosis may not be accurate; major changes in kyphosis were not seen.

## Conclusions

With the present study, we can conclude that the use of intermediate screws along with short-segment stabilization of single-level vertebral injury in the thoracolumbar region provides stability and early consolidation of vertebral bodies and maintains the mobility of spinal segment.
